# Plasmonic Waveguide Coupled Ring Cavity for a Non-Resonant Type Refractive Index Sensor

**DOI:** 10.3390/s17112526

**Published:** 2017-11-03

**Authors:** Soon-Hong Kwon

**Affiliations:** Department of Physics, Chung-Ang University, Seoul 06974, Korea; shkwon@cau.ac.kr; Tel.: +82-2-820-5844

**Keywords:** plasmonics, whispering gallery mode, cavity, refractive index sensor

## Abstract

Sensitive refractive index sensors with small footprints have been studied to allow the integration of a large number of sensors into a tiny chip for bio/chemical applications. In particular, resonant-type index sensors based on various micro/nanocavities, which use a resonant wavelength dependence on the refractive index of the analyte, have been developed. However, the spectral linewidth of the resonance, which becomes the resolution limit, is considerably large in plasmonic cavities due to the large absorption loss of metals. Therefore, there is demand for a new type of plasmonic refractive index sensor that is not limited by the linewidth of the cavity. We propose a new type of plasmonic index sensors consisting of a channel waveguide and a ring cavity. Two emissions from the ring cavity in both directions of the waveguide couple with a reflection phase difference depending on the length of a closed right arm with a reflecting boundary. Therefore, the output power dramatically and sensitively changes as a function of the refractive index of the analyte filling the waveguide.

## 1. Introduction

Biochemical optical sensors, which operate by measuring the resonant wavelength shift or a change of the output power of the sensor, must be able to measure a small refractive index change in a given liquid analyte. Many resonator-type refractive index sensors have been studied based on various dielectric microcavities such as photonic crystal cavities [1,2,3], microdisks [4], and microrings [5] because of their wavelength-scale compactness. These types of sensors can estimate a change in refractive index by measuring the wavelength shift of a resonance resulting from the refractive index change of the environmental analyte material surrounding the cavity. However, dielectric cavities have two limiting factors. One is that the confinement factor of the field in the analyte material is limited because a considerably large part of the electric field of the cavity mode exists in the dielectric material. In addition, the minimum physical size of the dielectric cavity is a half of the wavelength in the material’s diffraction limit, restricting further miniaturization of traditional cavity sensors.

In contrast, surface plasmon polaritons (SPPs) have been widely studied because of their ability to confine light in a subwavelength-sized region and to overcome the diffraction limit [6,7,8,9,10,11]. Since a large amount of energy exists in the environmental material in SPP sensors, they can more sensitively detect the change in refractive index. Recently, various plasmonic sensors have been reported using a variety of structures such as prism coupled resonators [12], nanoparticles [13,14], metamaterials [15], and cavities [16,17,18].

On the other hand, for resonator type sensors, it is important to reduce the linewidth of the resonance because the linewidth becomes the spectral resolution of the sensors, limiting the measurable index change [19]. In particular, any plasmonic cavities must have intrinsic metallic absorption losses, which result in linewidth broadening [20]. In order to overcome the linewidth limitation in plasmonic resonant index sensors, Mach-Zehnder interferometer (MZI) plasmonic sensors have been reported, which use the plasmonic waveguide mode of a metal-insulator-metal structure [21,22,23] or the long-range surface plasmon polariton mode of a metal stripe structure [24]. Such interferometer-type sensors use the change in output power resulting from the interference between two propagating waves. Therefore, they do not suffer the problem of having a small, but finite, linewidth that prevents the reduction of the detection limit. However, plasmonic MZI sensors require a relatively large size, namely, tens of micrometers, to provide sufficiently large path length differences [23].

In this paper, we propose a plasmonic index sensor consisting of a plasmonic ring cavity and a waveguide coupled with a mirror, which enables the detection of the index change by measuring the modulation of the output power. The output is determined by the interference between the left and right propagating waveguide modes coupled from the ring cavity; therefore, the proposed sensor does not suffer from the linewidth limit. In addition, due to the strong dependence of the waveguide mode on the refractive index, even at a small path length difference of 2 µm, a large normalized power sensitivity larger than 1600%/RIU (refractive index unit) can be achieved. The sensitivity of 1600%/RIU means that if the detector used in the proposed system can detect a 1% difference of in output power in the system, then a small index change of 1/1600 = 0.000625 can be measurable.

## 2. Concept of a Phase Sensitive Index Sensor

**Figure 1:**
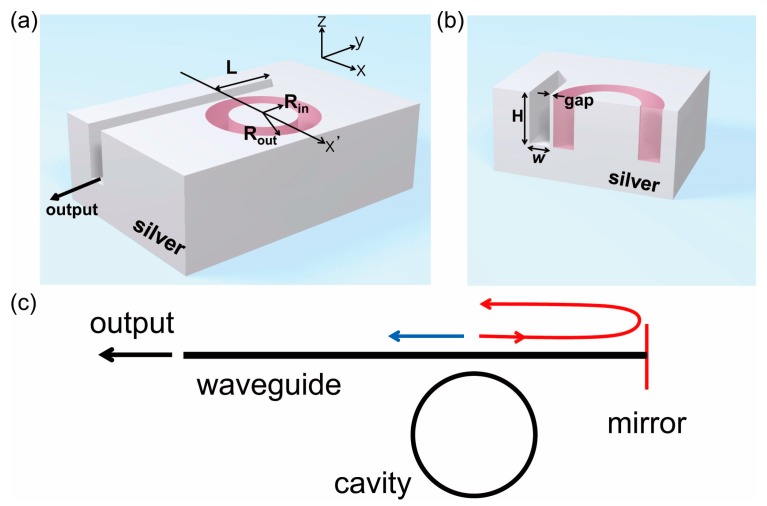
(**a**) A schematic diagram of the plasmonic ring cavity index sensor with a waveguide output port. The ring cavity (indicated by purple color) is filled by a light emitting material with a refractive index of 1.47. The waveguide and cavity, with rectangular cross-sections, are formed on the silver substrate. The right end of the waveguide is blocked by a silver wall, and the length to the right end from the coupling point is represented by *L*. All empty spaces including the waveguide are filled by an analyte. (**b**) A cross-sectional view of the ring cavity and waveguide along the *x*′ axis, indicated in Figure 1a at the coupling point. The waveguide has a groove with a rectangular cross-section with a width of *w* and a depth of *H* in the silver substrate. (**c**) A conceptual figure of a phase sensitive sensor. The blue and red arrows indicate the left and right propagating light coupled from the cavity into the waveguide, respectively.

We propose a plasmonic refractive index sensor consisting of a ring cavity and a waveguide formed on a silver substrate, where the refractive index of the analyte filling the waveguide can be measured by observing the output power from the output port of the waveguide, as shown in Figure 1a. The ring cavity, with an inner and outer radii of *R_in_* and *R_out_*, respectively, and a depth of *H*, is assumed to be filled by low index light emitting materials with an emission peak wavelength of 1550 nm, such as silica doped with erbium, which acts as a light source in the proposed sensor system operating by electrical or optical pumping. The waveguide, which couples light emitted from the ring cavity into the output ports, has an empty groove with a rectangular cross-section with a width of *w* and a depth of *H* on a silver substrate, as shown in Figure 1b. In all simulations, the waveguide and the free space outside of the ring cavity are filled with an analyte with a refractive index of 1.368. The ring cavity is separated by a thin silver wall at the coupling point. The left end of the waveguide is open to free space and is used to emit output power from the whole sensor system. The opposite end is blocked by a silver wall, which serves as a mirror for the right propagating wave, where the length from the coupling point to the mirror is denoted by *L*. The structure can be fabricated by depositing silver over a pre-fabricated molding of the ring cavity and the waveguide. Then, the chip is flipped, and the molding is removed from the waveguide by selective wet etching. The ring cavity light source can be fabricated by light emitting materials such as quantum dots embedded in PMMA (Polymethyl methacrylate). The proposed sensor can be integrated with microfluidic channels to introduce the analyte into the waveguide by similar approaches of the conventional microring resonator sensors [4,5]. However, in contrast to other sensors, optical or electrical pumping to the ring cavity light source should be considered.

Figure 1c shows the concept of the proposed refractive index sensor. From the ring cavity light source, the same amount of light is coupled into the left (blue) and right (red) propagating waves. If there is a mirror at the right side, as in Figure 1c, the right propagating light is reflected and couples with the left propagating light with accumulated phase differences. After the light enters the waveguide, the relative phase difference is proportional to the optical path length, which is the round-trip length (2*L*) over the right mirror multiplied by the change in propagation constant of the waveguide mode. If there is a change in refractive index in the waveguide and in the corresponding propagation constant, then the output power into free space can be largely modulated according to the phase difference. Therefore, the proposed refractive index sensor can measure the refractive index of the analyte filling the waveguide by observing the modulation of the output power.

## 3. Results and Discussions

**Figure 2:**
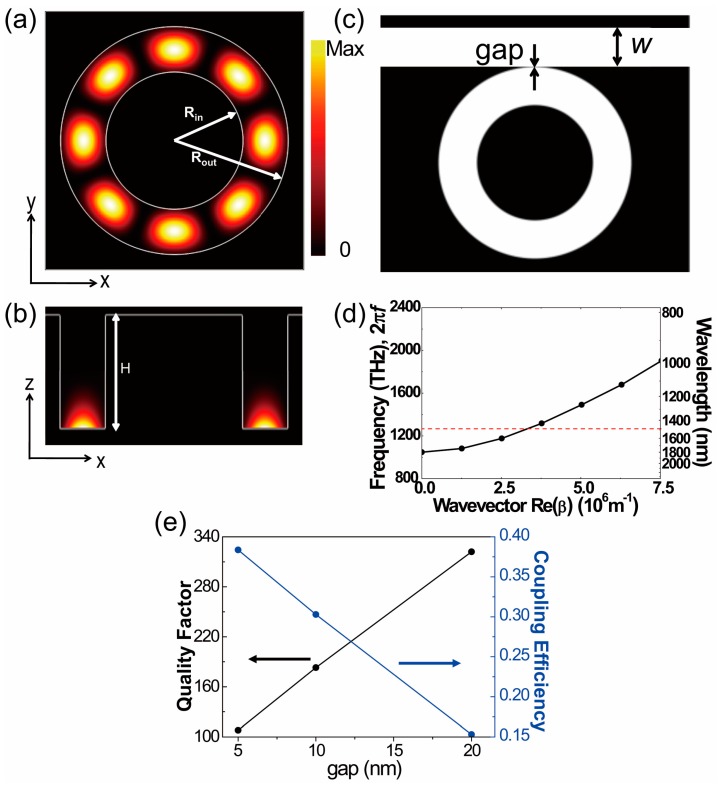
Electric field intensity profiles of the plasmonic whispering gallery mode in (**a**) top view and (**b**) cross-sectional view. The top view is achieved at the plane 20 nm above the bottom of the ring cavity. The outer and inner radii (*R_out_* and *R_in_*) of the ring cavity are 1500 nm and 900 nm, respectively. The depth (*H*) of the ring cavity is 1500 nm. (**c**) The waveguide with a width *w* of 600 nm is coupled with the ring cavity of Figure 2a. A silver wall with a gap thickness of 10 nm separates the waveguide and the cavity. (**d**) The dispersion curve of the rectangular groove waveguide with a width of 600 nm and a depth of 1500 nm. The red dotted line indicates 1518 nm, the resonant wavelength of the ring cavity. (**e**) The quality factor (black) and coupling efficiency (blue) as a function of gap thickness.

First, we calculated the plasmonic whispering gallery mode excited in the ring cavity by the three-dimensional (3D) finite-difference time-domain (FDTD) method. The software is home-built. The radii of the ring cavity, *R_in_* and *R_out_*, are 900 nm and 1500 nm, such that the width of the ring is 600 nm. The depth (*H*) is 1500 nm, which is deep enough to produce negligible radiation loss into free space. The electric field intensity profiles are shown in Figure 2a,b. The azimuthal mode number is 4, and the resonant wavelength is 1518 nm. Since the electric field maximum is placed at the bottom surface, as shown in Figure 2b, it is clear that the whispering gallery mode (WGM) is a surface plasmonic cavity mode.

On the other hand, plasmonic cavity loss can be divided into metallic absorption loss and radiation loss. The total quality (Q) factor is 464, and the metallic absorption loss is dominant (Q_absorption_ = 540). In contrast, the radiation loss is much smaller, and the corresponding Q_radiation_ is 3200, 7 times larger than the total Q factor. Here, the silver is modeled by the Drude model [25] and the refractive index of the ring cavity is set to 1.47. The Drude model is expressed as follows:

(1) $$\varepsilon (\omega )=\varepsilon_{\infty }-\frac{\omega_{D}^{2}}{\omega^{2}+i\gamma \omega }$$

Experimentally determined dielectric functions of silver were fitted by the background dielectric constant ($\varepsilon_{\infty }$) of 3.1, the plasma frequency ($\omega_{D}$) of 1.4 × 10^16^ s^−1^, and the collision frequency (γ) at room temperature of 3.1 × 10^13^ s^−1^, respectively. In order to represent infinite free space, the uniaxial perfectly matched layer (UPML) was used as the absorbing boundary condition. On the other hand, to calculate the dispersion curves (Figure 2d), a periodic boundary condition is used for the *y*-direction instead of the UPML boundary condition. In order to excite a ring cavity mode, an E_z_ dipole point source with a center wavelength of 1550 nm is placed 10 nm above the bottom of the ring cavity. A grid of 2.5 nm is used in the FDTD simulations.

Next, the ring cavity coupled with a waveguide without mirrors is considered for the light coupling into free space, as shown in Figure 2c. The waveguide width is chosen as 600 nm, which is the same width (600 nm) of the ring, *R_out_* − *R_in_*. The waveguide is separated by a silver wall width that is 10 nm thick.

In order to analyze the optical properties of the waveguide, the dispersion curve of the waveguide is investigated, as shown in Figure 2d. There is a cutoff frequency at the wavevector with a magnitude of zero, below which the propagation of the waveguide mode is forbidden. The resonant wavelength (1518 nm) of the ring cavity is indicated by the red dotted line, which exists on the dispersion curve of the waveguide. This means that the propagation of the waveguide mode, coupled from the ring cavity, is allowed. In addition, the propagation constant of the waveguide mode with a wavelength of 1518 nm corresponding the cavity resonance is $3.4\times 10^{6}$ m^−1^, and the surface plasmon wavelength is approximately 1840 nm.

As the gap size increases from 5 nm to 20 nm, the total Q factor increases, and the coupling efficiency decreases. Here, the coupling efficiency is defined as the ratio between the energy coupled into the waveguide and the total emitted output power from the ring cavity. For example, when the gap size is 20 nm, the coupling efficiency is 15% and the total Q factor is 322. At a gap of 10 nm, the Q factor decreases to 183 due to stronger light coupling to the waveguide, and the coupling efficiency is 30%. However, even in the case of a gap of 5 nm, the coupling efficiency slightly increases to 38% with a Q factor of 108. We chose 10 nm as a practical tradeoff between the increased coupling efficiency and reduced Q factor. Energy loss in the sensor system consisting of the ring cavity and the waveguide primarily originates from the intrinsic loss in the ring cavity, metallic absorption loss, and radiation loss.

**Figure 3:**
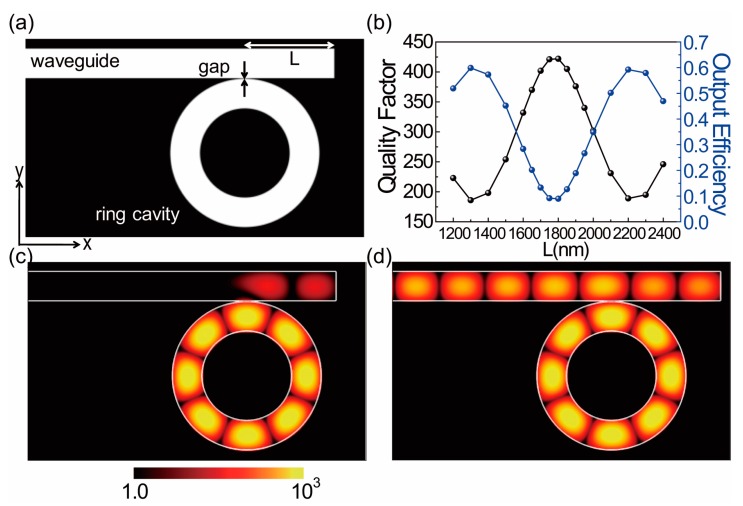
(**a**) A schematic diagram of the ring cavity and the coupled waveguide with a right end mirror. The length of the phase retardation from the coupling point to the right end mirror is indicated by *L*. The index of the material filling the waveguide is 1.368. (**b**) The quality factor (black) and output efficiency (blue) as a function of the length (*L*) of phase retardation. (**c**) Electric field intensity (log scale) profile at *L* = 1800 nm. The electric field intensity at the left output port of the waveguide is negligible. (**d**) The electric field intensity profile at *L* = 2200 nm. A strong output intensity is observed at the left port. The profiles of (**c**,**d**) are normalized by the maximum of the mode intensity of the ring cavity.

If the right end of the waveguide is blocked by a silver wall, as shown in Figure 3a, it acts as a good mirror for the propagating plasmonic waveguide mode. The scattering loss at the right end is negligible due to the large depth (*H*) of 1500 nm and the strong confinement of the plasmonic waveguide mode at the bottom surface of the waveguide. The gap size is set to 10 nm. The length (*L*) of the phase retardation for the right propagating plasmonic waveguide mode is defined from the coupling point to the right end of the waveguide. Here, the coupling point indicates the point where the waveguide is closest to the ring cavity. Due to the interference between the left and right propagating waveguide modes coupled to the cavity, as shown in Figure 1c, the output power can be modulated depending on the phase difference from the optical path length (*OL*) of the round trip of the right propagating mode, OL=β×2L. Therefore, if the propagation constant is unchanged, the retardation length (*L*) determines the degree of interference.

We investigated the quality factor and output efficiency of the system as a function of retardation length (*L*), as shown in Figure 3b. The Q factor of the system oscillates from a Q_max_ of 422 at *L* = 1800 nm to a Q_min_ of 189 at *L* = 2200 nm. In contrast, the output efficiency, defined as the ratio between the output power from the left end of the waveguide and the total emitted power from the ring cavity, has a minimum value of 9.0% at *L* = 1800 nm and maximum of 59% at *L* = 2200. The maximum of 59% is twice the coupling efficiency in the waveguide coupled from the ring cavity in the absence of a reflecting mirror, as shown in Figure 2e. As mentioned previously, these oscillations of the Q factor and output efficiency originate from the destructive and constructive interference of the left and right propagating waveguide mode. The period of oscillation is approximately 900 nm, which agrees well with one half of the plasmonic wavelength (920 nm) estimated from the dispersion curve of Figure 2d, which indicates that the oscillation arises from differences in the optical path length.

The interference nodes and maxima are clearly observable in the electric field intensity profiles for the minimum or maximum output efficiency in Figure 3c,d. Figure 3c shows destructive interference, and the output power in the left output port is barely observed. On the other hand, in the case of the constructive interference of Figure 3d, a strong output power signal can be observed in the left output port.

As shown in Figure 2e, some portion of the total emitted output power from the ring cavity is dissipated in the cavity itself by radiation loss into free space and metallic absorption loss. In order to enhance the modulation contrast of the maximum and minimum output powers, it should be further studied whether the cavity loss can be reduced by introducing a high index dielectric layer with a lower metal Ohmic loss for the hybrid plasmonic mode or through preventing radiation loss by using a metal cap on the ring cavity.

**Figure 4:**
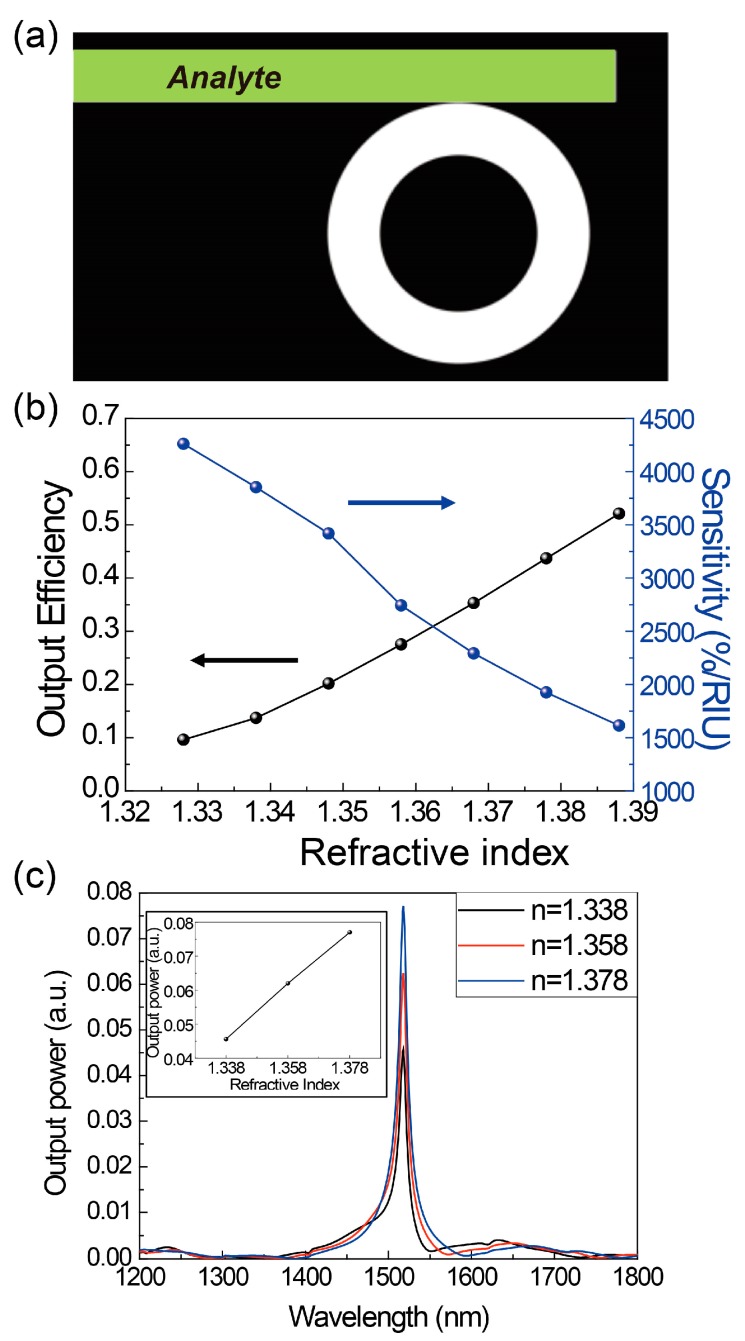
(**a**) A schematic diagram of the refractive index sensor that uses the proposed ring cavity system. The waveguide is filled with an analyte with a measurable refractive index. (**b**) The output efficiency (black) and normalized intensity sensitivity (blue) as a function of the refractive index to be measured. (**c**) The output power spectrum of a broadband dipole emitter for different refractive indices of the analyte: 1.338 (black), 1.358 (red), and 1.378 (blue). The dipole emitter is placed at one of the antinodes of the ring cavity. The inset shows the peak output power as a function of the refractive index.

According to the finding that the proposed ring cavity coupled with a waveguide with one mirror provides output power modulation that is sensitive to the optical path length (*OL*) of the wave retardation found in Figure 3, we propose a refractive index sensor where the refractive index of an analyte in the waveguide can be measured, as shown in Figure 4a, while the retardation length (*L*) is fixed to 2000 nm. Here, *L* = 2000 nm is chosen because the Q factor (the output efficiency) decreases (or increases) monotonically near this length. Since the optical phase difference from the coupling point to the right end mirror determines the interference, the change in propagation constant of the waveguide mode due to a change in refractive index [26] can induce modulation of the output power. Consequently, if output power modulation is monitored, the refractive index of the analyte can be measured.

In fact, the output efficiency linearly increases from 9% at *n* = 1.328 to 52% at *n* = 1.388, where the output power modulation is 43% for the small change in refractive index, *∆n*, of 0.06, as shown in Figure 4b. In order to estimate the performance of the index sensor, the normalized sensitivity *S*, defined by $S=\left(\frac{dI}{dn}\right)\frac{1}{I}\times 100\%$, is calculated for the refractive index range of interest, from 1.328 to 1.388, and has a recorded value larger than 1600%/RIU. In other words, if the detector in the index sensor system allows the detection of only a 1% change in output power of the system, a tiny index change of *∆n* = 1/1600 = 0.000625 can be measurable with the proposed system. Of course, in a practical device, the detection limit of the detector will determine the minimum measurable index change.

In order to investigate the spectral response, we also calculated the output power spectrum in a wide wavelength range from 1200 nm to 1800 nm for different refractive indices of the analyte: 1.338 (black), 1.358 (red), and 1.378 (blue), as shown in Figure 4c. A dipole emitter with a broad linewidth is placed horizontally at one of the antinodes of the ring cavity mode and vertically just above the bottom of the cavity, and the output power is obtained at the left end of the waveguide. One sharp output power peak with a wavelength of 1518 nm, the resonance of the ring cavity, is observed in a wide spectral range, having negligible wavelength shift for different indices, and the output power increases monotonically as the refractive index of the analyte increases (inset of Figure 4c). The normalized sensitivity is estimated as 1600%/RIU, as expected from Figure 4b. The linewidth of the spectral response is approximately 8 nm.

## 4. Conclusions

In this paper, we suggest a plasmonic index sensor based on a ring cavity with a diameter of 3 µm coupled to a waveguide with one mirror. For the path length difference of 2 µm, the output power largely modulates from 9% at *n* = 1.328 to 52% at *n* = 1.388 because of the strong interference between the left and right propagating waves. The normalized power sensitivity has a recorder value larger than 1600%/RIU, in which the detection limit of the refractive index is estimated as 0.000625 if the power detector can distinguish a 1% difference in output power. The proposed sensor, with a physical size of only several micrometers, can detect a small change in refractive index by monitoring the output power of the system, which can be used to develop a sensitive but high density integrated on-chip refractive index sensor.

The micron-scaled footprint of the proposed structure allows a good tolerance to thermal and mechanical fluctuations of the environments because spatial temperature or strain fluctuations can be neglected in a short scale sensor. The possible practical resolution of the structure is related to several key performances in the integrated sensor system, including the detection sensitivity and the power stability of the ring cavity light source. In contrast to that the detection sensitivity can be easily improved by using sensitive photodetectors, such as single photon avalanche photodiodes or photomuliplier tubes, the output power of the nanocavity light source can be unstable due to several reasons, such as small optical feedback from the system, changes in the Q factor of the cavity due to environmental index change, and fluctuations in electrical or optical pumping of the light source. Therefore, in order to develop practical and sensitive sensors by the proposed method, the power stability of the nanocavity light source should be also studied. Mach-Zehnder interferometers [27], ring resonators [28] and plasmonic interferometers [29] can have better detection limits of 7 × 10^−6^, 1 × 10^−5^, and 1 × 10^−5^, respectively. However, the footprints for these methods were a length of 30 mm, area of 100 µm^2^, and 10 × 30 µm^2^, which are tens of thousands to 20 times larger than the footprint of the proposed structure, 2 × 2 µm^2^. The proposed sensor has high sensitivity with a detection limit of 10^−4^ RIU and a small footprint of several micrometers. Therefore, it can be used as a portable liquid analyzer or a gas sensor [5], which require extremely small sensing volumes with relatively high sensitivity.

## References

[1] Kwon S.-H., Suenner T., Kamp M., Forchel A. (2008). Optimization of photonic crystal cavity for chemical sensing. Opt. Express.

[2] Suenner T., Stichel T., Kwon S.-H., Schlereth T.W., Hoefling S., Kamp M., Forchel A. (2007). Photonic crystal cavity based gas sensor. Appl. Phys. Lett..

[3] Jagerska J., Zhang H., Diao Z., Thomas N.L., Houdre R. (2010). Refractive index sensing with an air-slot photonic crystal nanocavity. Opt. Lett..

[4] Armani A.M., Vahala K.J. (2006). Heavy water detection using ultrahigh-Q microcavities. Opt. Lett..

[5] Robinson J.T., Chen L., Lipson M. (2008). On-chip gas detection in silicon optical microcavities. Opt. Express.

[6] Seo M.K., Kwon S.-H., Ee H.S., Park H.G. (2009). Full three-dimensional subwavelength high-Q surface-plasmon-polariton cavity. Nano Lett..

[7] Kang J.H., Park H.G., Kwon S.-H. (2011). Room-temperature high-Q channel-waveguide surface plasmon nanocavity. Opt. Express.

[8] Kang J.H., No Y.S., Kwon S.-H., Park H.G. (2011). Ultrasmall subwavelength nanorod plasmonic cavity. Opt. Lett..

[9] Kwon S.-H., Kang J.H., Seassal C., Kim S.K., Regreny P., Lee Y.H., Lieber C.M., Park H.G. (2010). Subwavelength plasmonic lasing from a semiconductor nanodisk with silver nanopan cavity. Nano Lett..

[10] Sorger V.J., Oulton R.F., Yao J., Bartal G., Zhang X. (2009). Plasmonic Fabry–Pérot nanocavity. Nano Lett..

[11] Kuttge M., Abajo F.J.G., Polman A. (2009). Ultrasmall mode volume plasmonic nanodisk resonators. Nano Lett..

[12] Shalabney A., Abdulhalim I. (2012). Figure-of-merit enhancement of surface plasmon resonance sensors in the spectral interrogation. Opt. Lett..

[13] Antosiewicz T.J., Apell S.P., Claudio V., Kall M. (2012). A simple model for the resonance shift of localized plasmons due to dielectric particle adhesion. Opt. Express.

[14] Anker J.N., Hall W.P., Lyandres O., Shah N.C., Zhao J., Van Duyne R.P. (2008). Biosensing with plasmonic nanosensors. Nat. Mater..

[15] Liu N., Mesch M., Weiss T., Hentschel M., Giessen H. (2010). Infrared perfect absorber and its application as plasmonic sensor. Nano Lett..

[16] Ameling R., Langguth L., Hentschel M., Mesch M., Braun P.V., Giessen H. (2010). Cavity-enhanced localized plasmon resonance sensing. Appl. Phys. Lett..

[17] Schmidt M.A., Lei D.Y., Wondraczek L., Nazabal V., Maier S.A. (2012). Hybrid nanoparticle–microcavity-based plasmonic nanosensors with improved detection resolution and extended remote-sensing ability. Nat. Commun..

[18] Kwon S.-H. (2013). Ultrasmall plasmonic cavity for chemical sensing. Plasmonics.

[19] White I.M., Fan X. (2008). On the performance quantification of resonant refractive index sensors. Opt. Express.

[20] Kwon S.-H. (2013). Deep subwavelength-scale metal-insulator-metal plasmonic disk cavities for refractive index sensors. IEEE Photonics J..

[21] Gan Q., Gao Y., Bartoli F.J. (2009). Vertical plasmonic Mach-Zehnder interferometer for sensitive optical sensing. Opt. Express.

[22] Gao Y., Gan Q., Xin Z., Cheng X., Bartoli F.J. (2011). Plasmonic Mach-Zehnder interferometer for ultrasensitive on-chip biosensing. ACS Nano.

[23] Zeng X., Gao Y., Hu H., Ji D., Gan Q., Bartoli F.J. (2013). A metal-insulator-metal plasmonic Mach-Zehnder interferometer array for multiplexed sensing. J. Appl. Phys..

[24] Khan A., Krupin O., Lisicka-Skrzek E., Berini P. (2013). Mach-Zehnder refractometric sensor using long-range surface plasmon waveguides. Appl. Phys. Lett..

[25] Johnson P.B., Christy R.W. (1972). Optical-constants of noble-metals. Phys. Rev. B.

[26] Moon K., Lee T.-W., Lee Y.J., Kwon S.-H. (2017). A metal-insulator-metal deep subwavelength cavity based on cutoff frequency modulation. Appl. Sci..

[27] Prieto F., Sepulveda B., Calle A., Llobera A., Dominguez C., Abad A., Montoya A., Lechuga L.M. (2003). An integrated optical interferometric nanodevice based on silicon technology for biosensor applications. Nanotechnology.

[28] De Vos K., Bartolozzi I., Schacht E., Bienstman P., Baets R. (2007). Silicon-on-insulator microring resonator for sensitive and label-free biosensing. Opt. Express.

[29] Gao Y., Xin Z., Gan Q., Cheng X., Bartoli F. (2013). Plasmonic interferometers for label-free multiplexed sensing. Opt. Express.

